# Effects of different positive end-expiratory pressure titration strategies during prone positioning in patients with acute respiratory distress syndrome: a prospective interventional study

**DOI:** 10.1186/s13054-022-03956-8

**Published:** 2022-03-26

**Authors:** Christoph Boesing, Peter T. Graf, Fabian Schmitt, Manfred Thiel, Paolo Pelosi, Patricia R. M. Rocco, Thomas Luecke, Joerg Krebs

**Affiliations:** 1 Department of Anaesthesiology and Critical Care Medicine, University Medical Centre Mannheim, Medical Faculty Mannheim of the University of Heidelberg, Theodor-Kutzer Ufer 1-3, 68167 Mannheim, Germany; 2 Department of Surgical Sciences and Integrated Diagnostics, University of Genoa, Genoa, Italy; 3 Anesthesiology and Critical Care - San Martino Policlinico Hospital, IRCCS for Oncology and Neurosciences, Genoa, Italy; 4 Laboratory of Pulmonary Investigation, Carlos Chagas Filho Institute of Biophysics, Federal University of Rio de Janeiro, Centro de Ciências da Saúde, Avenida Carlos Chagas Filho, 373, Bloco G-014, Ilha do Fundão, Rio de Janeiro, Brazil

**Keywords:** Acute respiratory distress syndrome, Positive end-expiratory pressure, Transpulmonary pressure, Respiratory mechanics, Prone position, Ventilator-induced lung injury, Lung protective ventilation

## Abstract

**Background:**

Prone positioning in combination with the application of low tidal volume and adequate positive end-expiratory pressure (PEEP) improves survival in patients with moderate to severe acute respiratory distress syndrome (ARDS). However, the effects of PEEP on end-expiratory transpulmonary pressure (Ptp_exp_) during prone positioning require clarification. For this purpose, the effects of three different PEEP titration strategies on Ptp_exp_, respiratory mechanics, mechanical power, gas exchange, and hemodynamics were evaluated comparing supine and prone positioning.

**Methods:**

In forty consecutive patients with moderate to severe ARDS protective ventilation with PEEP titrated according to three different titration strategies was evaluated during supine and prone positioning: (A) ARDS Network recommendations (PEEP_ARDSNetwork_), (B) the lowest static elastance of the respiratory system (PEEP_Estat,RS_), and (C) targeting a positive Ptp_exp_ (PEEP_Ptpexp_). The primary endpoint was to analyze whether Ptp_exp_ differed significantly according to PEEP titration strategy during supine and prone positioning.

**Results:**

Ptp_exp_ increased progressively with prone positioning compared with supine positioning as well as with PEEP_Estat,RS_ and PEEP_Ptpexp_ compared with PEEP_ARDSNetwork_ (positioning effect *p* < 0.001, PEEP strategy effect *p* < 0.001). PEEP was lower during prone positioning with PEEP_Estat,RS_ and PEEP_Ptpexp_ (positioning effect *p* < 0.001, PEEP strategy effect *p* < 0.001). During supine positioning, mechanical power increased progressively with PEEP_Estat,RS_ and PEEP_Ptpexp_ compared with PEEP_ARDSNetwork_, and prone positioning attenuated this effect (positioning effect *p* < 0.001, PEEP strategy effect *p* < 0.001). Prone compared with supine positioning significantly improved oxygenation (positioning effect *p* < 0.001, PEEP strategy effect *p* < 0.001) while hemodynamics remained stable in both positions.

**Conclusions:**

Prone positioning increased transpulmonary pressures while improving oxygenation and hemodynamics in patients with moderate to severe ARDS when PEEP was titrated according to the ARDS Network lower PEEP table. This PEEP titration strategy minimized parameters associated with ventilator-induced lung injury induction, such as transpulmonary driving pressure and mechanical power. We propose that a lower PEEP strategy (PEEP_ARDSNetwork_) in combination with prone positioning may be part of a lung protective ventilation strategy in patients with moderate to severe ARDS.

***Trial registration*:**

German Clinical Trials Register (DRKS00017449). Registered June 27, 2019. https://www.drks.de/drks_web/navigate.do?navigationId=trial.HTML&TRIAL_ID=DRKS00017449

**Supplementary Information:**

The online version contains supplementary material available at 10.1186/s13054-022-03956-8.

## Background

Severe acute respiratory distress syndrome (ARDS) is a life-threatening pulmonary disease characterized by inhomogeneous distribution of lung injury with alveolar consolidation/atelectasis and increased shunt fraction resulting in hypoxemia [1]. Positive pressure mechanical ventilation is a life-saving intervention but increases the risk of ventilator-induced lung injury (VILI) mediated by stress, strain, and energy transmission to the inflamed lung parenchyma [2]. Positive end-expiratory pressure (PEEP) increases end-expiratory transpulmonary pressure (Ptp_exp_), and prevent atelectasis, mainly in the dependent lung regions [3]. However, during protective mechanical ventilation, the optimal PEEP titration strategy remains controversial when taking into account the differences in lung recruitability of the individual patient [4]. Several strategies have been proposed to set PEEP [5]: (A) the use of a minimal PEEP level to achieve adequate oxygenation [6] according to the lower PEEP/fraction of inspired oxygen (FiO_2_) table, which was recommended by the ARDS Network [7]; (B) evaluation of the lowest static elastance of the respiratory system (*E*_stat,RS_) [8] aiming to achieve the lowest driving pressure (*P*_driv_), thus determining a compromise between recruitment and overinflation [9, 10]; and (C) targeting a positive Ptp_exp_ to account for variability in lung and chest wall mechanics and optimize alveolar recruitment [11]. The pleural pressure is increased in dependent lung regions leading to alveolar collapse [12], therefore the application of a matched PEEP should counteract the pleural pressure and thus promote the balance between recruitment and overdistension [13]. In addition, prone positioning is an effective strategy in patients with moderate to severe ARDS and is known to decrease mortality [14]. This decrease is likely due to the more homogeneous distribution of ventilation as a result of reduced vertical pleural pressure gradient [15], which may lead to less lung damage [16, 17] and improved lung mechanics. To date, the interaction between prone positioning and Ptp_exp_ when using different PEEP titration strategies has not been clarified [18, 19]. We hypothesized that Ptp_exp_ differed significantly according to PEEP titration strategy during supine and prone positioning in patients with moderate to severe ARDS. Secondary endpoints were to evaluate the effects of different PEEP titration strategies on respiratory system, lung and chest wall mechanics, mechanical power, gas exchange and hemodynamics during supine and prone positioning.

## Methods

This prospective interventional study was conducted from July 2019 to February 2021 with approval from the local ethical committee (Medizinische Ethikkomission II, University Medical Centre Mannheim, Medical Faculty Mannheim of the University of Heidelberg, Mannheim, registration number 2018-609N-MA) and study registration at the German Clinical Trials Register (DRKS00017449) in the intensive care unit (ICU) of the Department of Anesthesiology and Critical Care Medicine, University Medical Centre Mannheim, Medical Faculty Mannheim of the University of Heidelberg in Mannheim, Germany. All mechanically ventilated patients in the ICU were screened for the presence of moderate to severe ARDS (defined by the ratio of arterial oxygen partial pressure divided by the fraction of inspired oxygen [PaO_2_/FiO_2_] < 150 mm Hg) [20]. After obtaining written informed consent from each patient or their relatives, 40 consecutive patients with moderate to severe ARDS (defined by the ratio of arterial oxygen partial pressure divided by the fraction of inspired oxygen [PaO_2_/FiO_2_] < 150 mm Hg) were studied. Exclusion criteria were age younger than 18 years, pregnancy, end-stage chronic organ failure, inherited cardiac malformations, severe head injury and hemodynamic instability (mean arterial pressure [MAP] < 65 mm Hg, cardiac index [CI] of < 2.0 L/min/m^2^).

All patients were ventilated with an Engström Carescape™ R860 ventilator (GE Healthcare, Munich, Germany) and had a five-lumen central venous catheter inserted via the internal jugular vein for the measurement of central venous pressure (CVP) and central venous oxygen saturation (S_cv_O_2_). A thermodilution catheter (4F or 5F Pulsiocath™, Pulsion Medical Systems, Munich, Germany) was inserted in the femoral artery to allow hemodynamic measurement and fluid management through a Pulse Contour Cardiac Output monitor (PiCCOplus™, Pulsion Medical Systems, Munich, Germany). Esophageal pressure was measured with an esophageal balloon catheter (NutriVent™ nasogastric catheter, Sidam, Mirandola, Italy) filled with 4 mL of air as indicated by the manufacturer. The esophageal balloon catheter was positioned by slow retraction from the stomach until maximal respiratory pressure swings and minimal cardiac oscillation artefacts were obtained. Catheter positioning was confirmed by applying manual compression on the chest during an end-expiratory airway occlusion. Correct positioning was verified by a ratio of change in esophageal pressure to the change in airway pressure of 0.8–1.2 [21]. All patients were sedated with midazolam (5–15 mg/h) and sufentanil (30–40 µg/h) to achieve a Richmond Agitation-Sedation Score of − 5 [22]. Cisatracurium was infused continuously for neuromuscular blockade throughout the study period. Norepinephrine was administered if MAP was below 65 mmHg despite preload optimization. The patients were ventilated in a volume-controlled mode with tidal volumes (*V*_T_) of 6 mL/kg predicted body weight and respiratory rates (RR) to achieve a pHa of 7.25. In accordance with the study protocol, PEEP was initially set using the ARDS Network lower PEEP table (PEEP_ARDSNetwork_) [7]. Allowable combinations of FiO_2_ and PEEP are presented in Additional file 1: Table S1.

## Experimental protocol

**Fig. 1:**
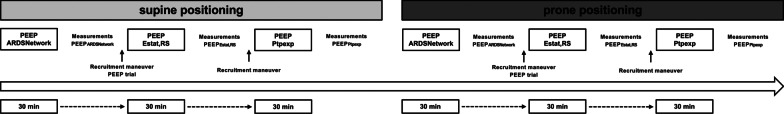
Schematic workflow of the study. *PEEP* positive end-expiratory pressure, *PEEP*_*ARDSNetwork*_ PEEP titrated according to the ARDS Network lower PEEP table, *PEEP*_*Estat,RS*_ PEEP titrated according to the lowest elastance of the respiratory system, *PEEP*_*Ptpexp*_ PEEP titrated according to end-expiratory transpulmonary pressure

The schematic workflow of the study is presented in Fig. 1. After complete measurement of respiratory mechanics, gas exchange, and hemodynamics at PEEP_ARDSNetwork_, a standardized dynamic recruitment maneuver and a decremental PEEP trial was performed (Additional file 1: Figure S1).

PEEP was slowly increased to 35 cm H_2_O in a pressure-controlled mode with a *P*_driv_ of 15 cm H_2_O over a period of 5 min. After 2 min, ventilator mode was switched back to a volume-controlled mode using the initial *V*_T_ and RR to perform a decremental PEEP trial. Starting with a PEEP of 30 cm H_2_O, the PEEP was reduced stepwise by 2 cm H_2_O every 2 min until *E*_stat,RS_ did not decrease further with a reduction of PEEP. The identified PEEP with the lowest *E*_stat,RS_ (PEEP_Estat,RS_) was set after a recruitment maneuver, and a complete measurement of respiratory mechanics, gas exchange, and hemodynamics was performed after a 30-min equilibration period. After a recruitment maneuver as described, PEEP was set to the Ptp_exp_ target according to the empirical table of the EPVent-2 trial [23] (PEEP_Ptpexp_). Allowable combinations of FiO_2_ and Ptp_exp_ are presented in Additional file 1: Table S2. After a 30-min equilibration period, complete measurement of respiratory mechanics, gas exchange, and hemodynamics was performed. Patients were then moved to the prone position and all the physiological measurements were repeated with PEEP_ARDSNetwork_ followed by another titration of PEEP_Estat,RS_ and PEEP_Ptpexp_ as described above.

### Respiratory mechanics, gas exchange and hemodynamics

Respiratory mechanics, gas exchange and hemodynamics were obtained following the equilibration period for PEEP_ARDSNetwork_, PEEP_Estat,RS_ and PEEP_Ptpexp_ in supine and prone position. The mechanics of the respiratory system, lung and chest wall were calculated according to the standard formulas (see Additional file 1). End-inspiratory esophageal pressure (Pes_insp_) and end-expiratory esophageal pressure (Pes_exp_) were recorded during a 5-s inspiratory and 5-s expiratory hold, respectively. The mechanical power, the ratio of physiologic dead space to tidal volume (*V*_D_/*V*_T_) and the ventilatory ratio were computed according to the conventional equations (see Additional file 1). End-expiratory lung volume (EELV) was measured with the Engström Carescape™ R860 ventilator using the nitrogen wash-in/wash-out technique [24]. All hemodynamic parameters were obtained using the Pulse Contour Cardiac Output monitor after calibration with the transpulmonary thermodilution method using 20 mL iced saline three times. Blood gas analyses were made with a blood gas analyzer (Radiometer ABL 800 Flex, Radiometer, Willich, Germany). For the measurement of intraabdominal pressure (IAP), the transducer was zeroed at the level of the midaxillary line at end-expiration using an instillation volume of 25 mL of saline in the bladder as recommended by the Abdominal Compartment Society [25]. The Simplified Acute Physiology Score II (SAPS II) [26] and Sequential Organ Failure Assessment (SOFA) score [27] were calculated for each patient on admission to the intensive care unit.

### Statistical analysis

The primary end point was to analyze Ptp_exp_ when PEEP was set according to three different strategies both in supine and prone positioning in patients with moderate to severe ARDS. Secondary endpoints were to evaluate the effects of different PEEP titration strategies on respiratory system, lung and chest wall mechanics, mechanical power, gas-exchange, and hemodynamics during supine and prone positioning. The number of patients was calculated based on a previous study conducted by our group [28], in which we assumed that the expected value of Ptp_exp_ at PEEP titrated to the lowest *E*_stat,RS_ is zero with a standard deviation of 4.40 in the supine position. For the prone position, we assumed the same standard deviation but an expected value of Ptp_exp_ equal to 3 cm H_2_O. We further assumed that the correlation between both measures would be non-negative. Therefore, under these conditions, a sample size of 40 patients showed a power higher than 80% for a two-way repeated measurement ANOVA, with a significance level of 5%.

Statistical analysis was performed using SigmaPlot 12.5 (Systat Software GmbH, Erkrath, Germany). Categorical variables were compared using Fisher’s exact test and presented as frequency and percentages. For continuous variables, the normality of the data and the homogeneity of variances were tested using the Shapiro–Wilk test and Levene’s median test, respectively. As per the study protocol, longitudinal physiologic data were analyzed using two-way repeated measures ANOVA followed by Holm-Sidak’s post hoc test. The results are expressed as means ± standard deviation. The level of significance was set at *p* < 0.05.

## Results

Forty consecutively identified patients with moderate to severe ARDS (PaO_2_/FiO_2_ < 150) were included in the analysis. Demographic and clinical characteristics of the patients are shown in Additional file 1, Table S3.

### Transpulmonary pressures

**Fig. 2:**
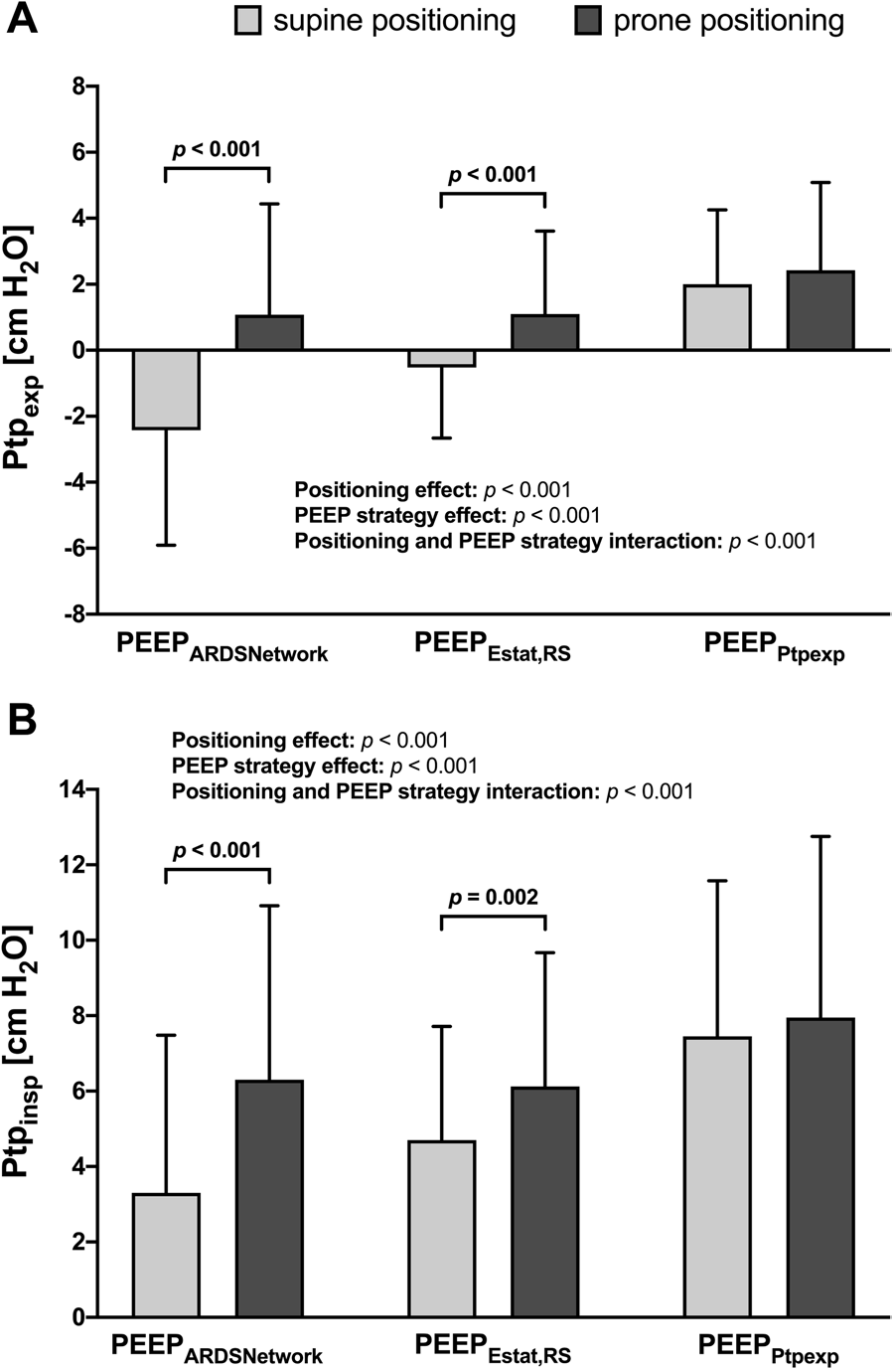
Effects of different PEEP titration strategies on Ptp_exp_ and Ptp_insp_ during supine and prone positioning. **A** Ptp_exp_ evaluated according to different PEEP titration strategies during supine and prone positioning. **B** Ptp_insp_ evaluated according to different PEEP titration strategies during supine and prone positioning. Bars are means + SD of 40 patients with moderate to severe ARDS. Brackets denote statistically significant differences between PEEP titration strategies, *p*-values are shown above the brackets. *PEEP* positive end-expiratory pressure, *PEEP*_*ARDSNetwork*_ PEEP titrated according to the ARDS Network lower PEEP table, *PEEP*_*Estat,RS*_ PEEP titrated according to the lowest elastance of the respiratory system, *PEEP*_*Ptpexp*_ PEEP titrated according to end-expiratory transpulmonary pressure, *Ptp*_*exp*_ end-expiratory transpulmonary pressure, *Ptp*_*insp*_ end-inspiratory transpulmonary pressure

Prone compared to supine positioning increased end-expiratory transpulmonary pressure (Ptp_exp_) when using PEEP_ARDSNetwork_ (− 2.4 ± 3.5 versus 1.1 ± 3.4 cm H_2_O, *p* < 0.001) and PEEP_Estat,RS_ (− 0.5 ± 2.1 versus 1.1 ± 2.5 cm H_2_O, *p* < 0.001) (Fig. 2A). End-inspiratory transpulmonary pressure (Ptp_insp_) also increased using PEEP_ARDSNetwork_ (3.3 ± 4.2 *versus* 6.3 ± 4.6 cm H_2_O, *p* < 0.001) and PEEP_Estat,RS_ (4.7 ± 3.0 versus 6.1 ± 3.5 cm H_2_O, *p* < 0.001) (Fig. 2B). There was a significant effect of positioning and PEEP strategy as well as an interaction between position and PEEP strategy for Ptp_exp_ and Ptp_insp_ (*p* < 0.001 each). Prone positioning reduced transpulmonary driving pressure (Ptp_driv_) when using PEEP_ARDSNetwork_ (5.7 ± 2.2 versus 5.2 ± 2.1 cm H_2_O, *p* = 0.031) (Fig. 4B).

### Respiratory mechanics

**Fig. 3:**
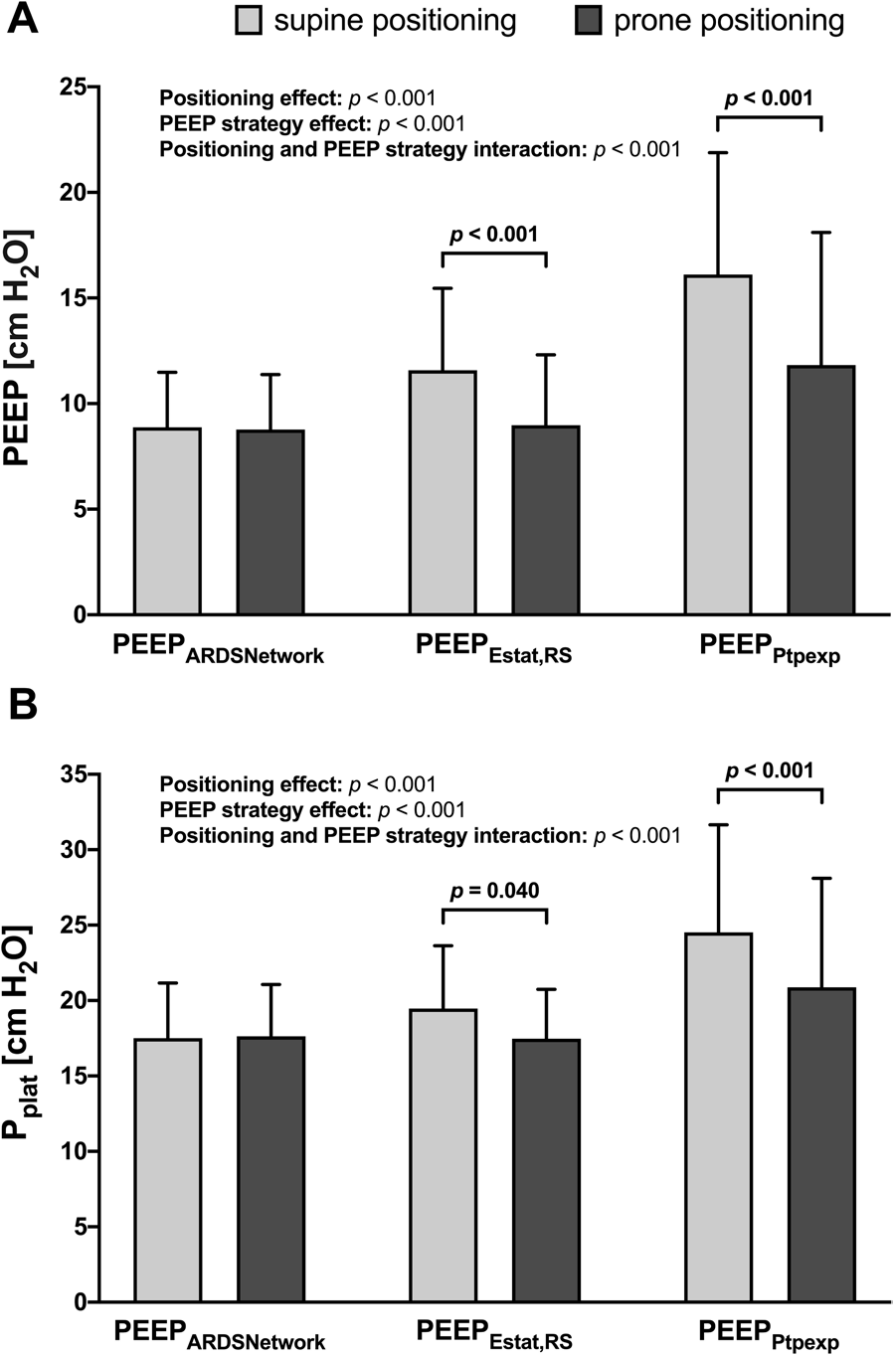
Effects of different PEEP titration strategies on PEEP and *P*_plat_ during supine and prone positioning. **A** PEEP setting according to different PEEP titration strategies during supine and prone positioning. **B** *P*_plat_ evaluated according to different PEEP titration strategies during supine and prone positioning. Bars are means + SD of 40 patients with moderate to severe ARDS. Brackets denote statistically significant differences between PEEP titration strategies strategies, *p*-values are shown above the brackets. *PEEP* positive end-expiratory pressure, *PEEP*_*ARDSNetwork*_ PEEP titrated according to the ARDS Network lower PEEP table, *PEEP*_*Estat,RS*_ PEEP titrated according to the lowest elastance of the respiratory system, *PEEP*_*Ptpexp*_ PEEP titrated according to end-expiratory transpulmonary pressure, *P*_*plat*_ end-inspiratory plateau airway pressure

The titrated PEEP levels were lower in the prone position compared with the supine position using PEEP_Estat,RS_ (11.6 ± 3.9 versus 9.0 ± 3.3 cm H_2_O, *p* < 0.001) and PEEP_Ptpexp_ (16.1 ± 5.8 versus 11.8 ± 6.3 cm H_2_O, *p* < 0.001) but not for PEEP_ARDSNetwork_ (Fig. 3A). Similarly, in the prone position, end-inspiratory plateau airway pressure (*P*_plat_) decreased using PEEP_Estat,RS_ (19.5 ± 4.2 versus 17.5 ± 3.3 cm H_2_O, *p* = 0.04) and PEEP_Ptpexp_ (24.5 ± 7.1 versus 20.9 ± 7.2 cm H_2_O, *p* < 0.001) (Fig. 3B). PEEP and *P*_plat_ differed significantly according to the position (*p* < 0.001) and PEEP strategy (*p* < 0.001). There was an interaction between position and PEEP strategy for PEEP and *P*_plat_ (*p* < 0.001).

**Fig. 4:**
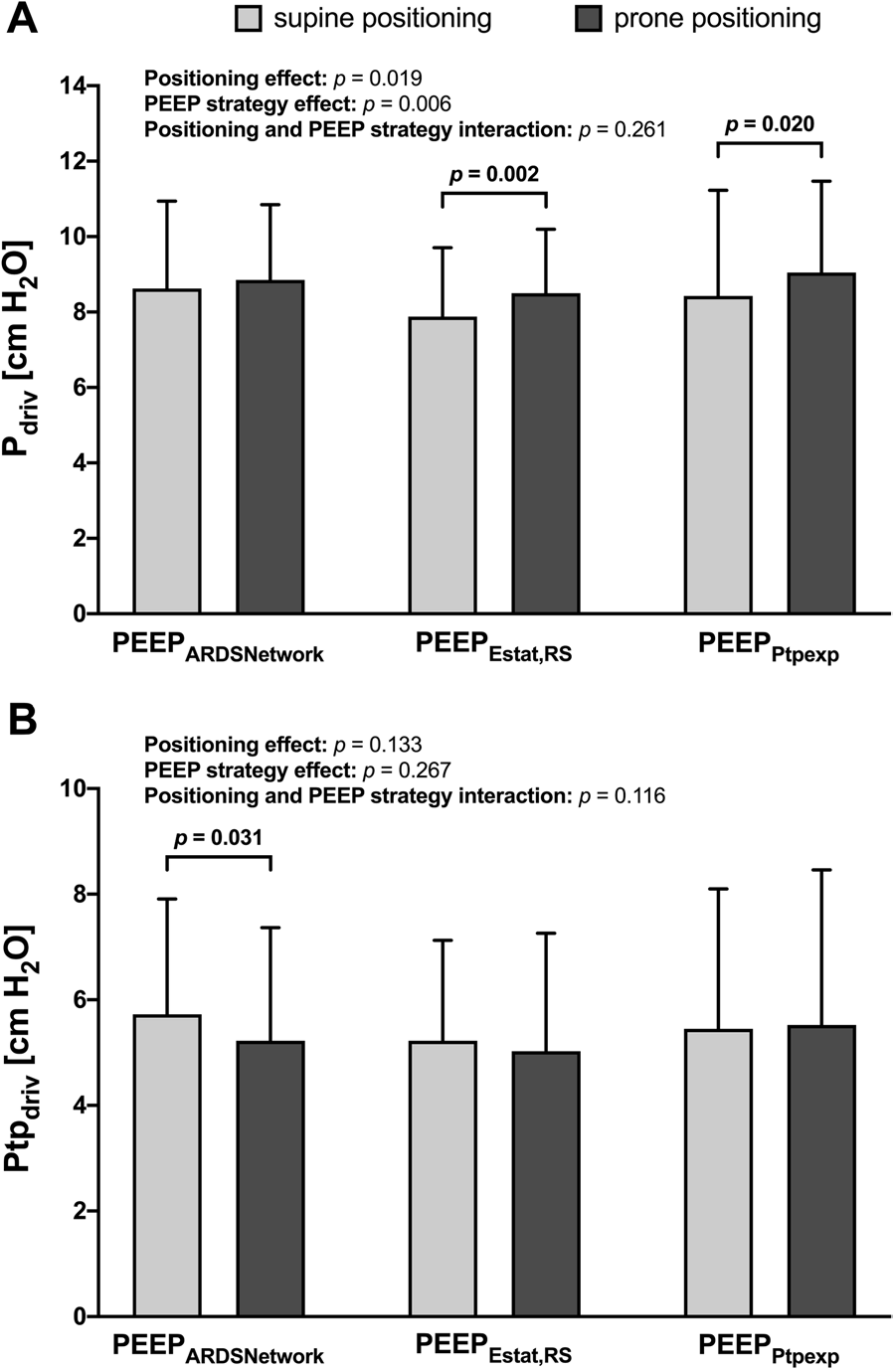
Effects of different PEEP titration strategies on *P*_driv_ and Ptp_driv_ during supine and prone positioning. **A** *P*_driv_ evaluated according to different PEEP titration strategies during supine and prone positioning. **B** Ptp_driv_ evaluated according to different PEEP titration strategies during supine and prone positioning. Bars are means + SD of 40 patients with moderate to severe ARDS. Brackets denote statistically significant differences between PEEP titration strategies strategies, *p*-values are shown above the brackets. *P*_*driv*_ driving pressure, *PEEP* positive end-expiratory pressure, *PEEP*_*ARDSNetwork*_ PEEP titrated according to the ARDS Network lower PEEP table, *PEEP*_*Estat,RS*_ PEEP titrated according to the lowest elastance of the respiratory system, *PEEP*_*Ptpexp*_ PEEP titrated according to end-expiratory transpulmonary pressure, *Ptp*_*driv*_ transpulmonary driving pressure pressure

In the prone position, driving pressure (*P*_driv_) increased using PEEP_Estat,RS_ (7.9 ± 1.8 versus 8.5 ± 1.7 cm H_2_O, *p* = 0.002) and PEEP_Ptpexp_ (8.4 ± 2.8 versus 9.1 ± 2.4 cm H_2_O, *p* = 0.02) (Fig. 4A). The effects of positioning (*p* = 0.019) and PEEP strategy (*p* = 0.006) were significant for *P*_driv_.

Mechanical power decreased using PEEP_Estat,RS_ (19.2 ± 5.9 versus 17.5 ± 5.8 J/min, *p* < 0.001) and PEEP_Ptpexp_ (24.4 ± 9.8 versus 21.0 ± 9.6 J/min, *p* < 0.001) in the prone position. Mechanical power differed significantly according to the position (*p* < 0.001), PEEP strategy (*p* < 0.001) and interaction between positioning and PEEP strategy (*p* < 0.001) (Table 1).

**Table 1:** Respiratory mechanics using three different PEEP titration strategies during supine and prone positioning

	PEEP_ARDSNetwork_		PEEP_Estat,RS_		PEEP_Ptpexp_		*p* values		
	Supine	Prone	Supine	Prone	Supine	Prone	Positioning effect	PEEP strategy effect	Positioning and PEEP strategy interaction
RR (breaths/min)	22.3 ± 1.9	22.3 ± 1.9	22.3 ± 1.9	22.3 ± 1.9	22.3 ± 1.9	22.3 ± 1.9	1.000	1.000	1.000
*V*_T_ (mL/kg PBW)	6.2 ± 0.3	6.2 ± 0.3	6.2 ± 0.3	6.2 ± 0.3	6.2 ± 0.3	6.2 ± 0.3	1.000	1.000	1.000
*P*_peak,RS_ (cm H_2_O)	23.3 ± 5.1	23.5 ± 4.5	25.0 ± 5.1	23.3 ± 4.5*	30.6 ± 8.8	27.2 ± 8.8*	**< 0.001**	**< 0.001**	**< 0.001**
*P*_mean,RS_ (cm H_2_O)	13.5 ± 2.9	13.5 ± 2.7	15.8 ± 4.0	13.6 ± 3.4*	20.5 ± 6.1	16.5 ± 6.5*	**< 0.001**	**< 0.001**	**< 0.001**
Pes_insp_ (cm H_2_O)	14.2 ± 3.5	11.4 ± 3.7*	14.8 ± 4.4	11.4 ± 4.6*	17.1 ± 5.7	13.0 ± 6.0*	**< 0.001**	**< 0.001**	**0.007**
Pes_exp_ (cm H_2_O)	11.3 ± 3.1	7.7 ± 3.2*	12.1 ± 4.0	7.9 ± 4.0*	13.4 ± 5.4	9.4 ± 5.4*	**< 0.001**	**< 0.001**	0.572
∆Pes (cm H_2_O)	2.9 ± 1.6	3.6 ± 1.9*	2.7 ± 1.5	3.5 ± 1.9*	3.0 ± 1.8	3.5 ± 2.0*	**< 0.001**	0.141	0.339
*E*_stat,RS_ (cm H_2_O/L)	21.3 ± 6.5	21.7 ± 6.0	19.5 ± 5.8	20.8 ± 5.2*	20.7 ± 8.0	22.1 ± 6.4*	**0.032**	**0.023**	0.255
*E*_stat,CW_ (cm H_2_O/L)	7.1 ± 4.2	9.0 ± 5.0*	6.6 ± 4.2	8.6 ± 4.9*	7.3 ± 4.8	8.8 ± 5.2*	**< 0.001**	0.132	0.397
*E*_stat,L_ (cm H_2_O/L)	14.0 ± 5.5	13.0 ± 5.3	12.8 ± 4.8	12.6 ± 5.4	13.4 ± 6.9	13.9 ± 6.8	0.565	0.093	0.087
Mechanical power (J/min)	17.5 ± 5.7	17.6 ± 5.5	19.2 ± 5.9	17.5 ± 5.8*	24.4 ± 9.8	21.0 ± 9.6*	**< 0.001**	**< 0.001**	**< 0.001**
IAP (cm H_2_O)	8.3 ± 2.8	11.1 ± 3.0*	8.7 ± 2.9	11.1 ± 3.5*	9.9 ± 3.5	12.1 ± 3.8*	**< 0.001**	**< 0.001**	0.238
*V*_D_/*V*_T_ (%)	28.5 ± 10.9	25.2 ± 9.0*	27.6 ± 10.8	24.7 ± 9.5*	28.4 ± 10.5	25.8 ± 9.5*	**< 0.001**	0.224	0.739
Ventilatory rate	2.09 ± 0.5	2.11 ± 0.5	2.12 ± 0.5	2.13 ± 0.5	2.13 ± 0.5	2.14 ± 0.5	0.308	0.176	0.345
EELV (mL)	1630 ± 552	1972 ± 693*	1920 ± 556	1993 ± 627	2140 ± 615	2108 ± 736	**0.011**	**< 0.001**	**< 0.001**

Values are means ± standard deviation of 40 patients with moderate to severe ARDS. Two-way repeated measures ANOVA was used to compare the effects of different PEEP titration strategies on respiratory mechanics during supine and prone positioning (*p* < 0.05)

Bold numbers represent statistically significant differences between groups

*∆Pes* difference between esophageal pressure at plateau airway pressure and positive end-expiratory pressure, *EELV* end-expiratory lung volume, *E*_*stat,CW*_ static elastance of the chest wall, *E*_*stat,L*_ static elastance of the lung, *E*_*stat,RS*_ static elastance of the respiratory system, *IAP* intraabdominal pressure, *PBW* predicted body weight, *PEEP* positive end-expiratory pressure, *PEEP*_*ARDSNetwork*_ PEEP titrated according to the ARDS Network lower PEEP table, *PEEP*_*Estat,RS*_ PEEP titrated according to the lowest elastance of the respiratory system, *PEEP*_*Ptpexp*_ PEEP titrated according to end-expiratory transpulmonary pressure, *Pes*_*exp*_ esophageal pressure at positive end-expiratory pressure, *Pes*_*insp*_ esophageal pressure at plateau airway pressure, *P*_*mean,RS*_ mean airway pressure of the respiratory system, *P*_*peak,RS*_ peak airway pressure of the respiratory system, *RR* respiratory rate, *V*_*D*_/*V*_*T*_ ratio of physiologic dead space to tidal volume, *V*_*T*_ tidal volume

*Significant differences at each PEEP titration strategy between supine and prone positioning

End-expiratory lung volume (EELV) increased using PEEP_ARDSNetwork_ (1630 ± 552 versus 1972 ± 693 mL, *p* = 0.026) in the prone compared to the supine position. EELV differed significantly according to the position (*p* = 0.011), PEEP strategy (*p* < 0.001), and interaction between positioning and PEEP strategy (*p* < 0.001) (Table 1).

### Gas exchange and hemodynamics

Prone compared to supine positioning improved oxygenation regardless of the PEEP strategy (PaO_2_/FiO_2_: 136 ± 36 versus 228 ± 86 mm Hg in PEEP_ARDSNetwork_, *p* < 0.001; 170 ± 72 versus 237 ± 91 mm Hg in PEEP_Estat,RS_, *p* = 0.002 and 192 ± 76 versus 240 ± 100 mm Hg in PEEP_Ptpexp_, *p* = 0.002) (Table 2). Mean arterial pressure was higher in the prone position compared with the supine position independent of the PEEP strategy (83.0 ± 10.8 versus 87.1 ± 11.2 mm Hg in PEEP_ARDSNetwork_, *p* = 0.005; 82.6 ± 9.8 versus 89.5 ± 11.9 in PEEP_Estat,RS_, *p* < 0.001 and 79.2 ± 11.3 versus 87.6 ± 11.4 in PEEP_Ptpexp_, *p* < 0.001) (Table 2). Cardiac index was increased using PEEP_Estat,RS_ (3.5 ± 0.9 versus 3.7 ± 1.0 L/min/m^2^, *p* = 0.021) and PEEP_Ptpexp_ (3.2 ± 0.7 versus 3.6 ± 0.8 L/min/m^2^, *p* < 0.001) during prone positioning (Table 2).

**Table 2:** Gas exchange and hemodynamics using three different PEEP titration strategies during supine and prone positioning

	PEEP_ARDSNetwork_		PEEP_Estat,RS_		PEEP_Ptpexp_		*p* values		
	Supine	Prone	Supine	Prone	Supine	Prone	Positioning effect	PEEP strategy effect	Positioning and PEEP strategy interaction
PaO_2_/FiO_2_ (mm Hg)	136 ± 36	228 ± 86*	170 ± 72	237 ± 91*	192 ± 76	240 ± 100*	**< 0.001**	**< 0.001**	**< 0.001**
PaCO_2_ (mm Hg)	57.0 ± 10.5	57.9 ± 10.5	57.6 ± 10.3	57.7 ± 10.7	57.6 ± 10.4	58.0 ± 10.4	0.346	0.308	0.297
pHa	7.3 ± 0.1	7.3 ± 0.1	7.3 ± 0.1	7.3 ± 0.1	7.3 ± 0.1	7.3 ± 0.1	0.812	0.070	0.101
HR (beats/min)	92.6 ± 19.1	93.1 ± 21.2	92.8 ± 20.5	93.2 ± 21.4	92.6 ± 19.2	92.4 ± 20.6	0.903	0.603	0.711
MAP (mm Hg)	83.0 ± 10.8	87.1 ± 11.2*	82.6 ± 9.8	89.5 ± 11.9*	79.2 ± 11.3	87.6 ± 11.4*	**< 0.001**	**0.009**	**0.011**
Norepinephrine (µg/kg/min)	0.2 ± 0.3	0.2 ± 0.3	0.2 ± 0.3	0.2 ± 0.3	0.2 ± 0.2	0.2 ± 0.2	0.182	0.458	0.756
CVP (mm Hg)	14.1 ± 6.4	16.8 ± 5.6*	15.1 ± 6.9	16.8 ± 6.2	16.4 ± 7.4	17.5 ± 6.8	0.068	**< 0.001**	**< 0.001**
S_cv_O_2_ (%)	75.3 ± 7.4	81.2 ± 6.8	77.6 ± 5.2	82.8 ± 6.2	77.6 ± 6.1	81.4 ± 6.3	**< 0.001**	**0.002**	**0.046**
CI (L/min/m^2^)	3.7 ± 1.0	3.7 ± 0.9	3.5 ± 0.9	3.7 ± 1.0*	3.2 ± 0.7	3.6 ± 0.8*	**0.004**	**< 0.001**	**< 0.001**

Values are means ± standard deviation of 40 patients with moderate to severe ARDS. Two-way repeated measures ANOVA was used to compare the effects of different PEEP titration strategies on gas exchange and hemodynamic parameters during supine and prone positioning (*p* < 0.05)

Bold numbers represent statistically significant differences between groups

*CI* cardiac index, *CVP* central venous pressure, *HR* heart rate, *MAP* mean arterial pressure, *PaCO*_*2*_ arterial partial pressure of carbon dioxide, *PEEP* positive end-expiratory pressure, *PEEP*_*ARDSNetwork*_ PEEP titrated according to the ARDS Network lower PEEP table, *PEEP*_*Estat,RS*_ PEEP titrated according to the lowest elastance of the respiratory system, *PEEP*_*Ptpexp*_ PEEP titrated according to end-expiratory transpulmonary pressure, *PaO*_*2*_*/FiO*_*2*_ arterial oxygen partial pressure divided by the fraction of inspired oxygen, *pHa* negative logarithm of the molar concentration of dissolved hydronium ions in arterial blood, *S*_*cv*_*O*_*2*_ central venous oxygen saturation

*Significant differences at each PEEP titration strategy between supine and prone positioning

Further details regarding the effects of the three different PEEP titration strategies on respiratory mechanics, gas exchange and hemodynamics during supine and prone positioning are presented in Tables 1, 2, and Additional file 1: Table S4 to S9.

## Discussion

In patients with moderate to severe ARDS under protective mechanical ventilation, the interaction between three different PEEP titration strategies, positioning, and the resulting Ptp_exp_ was evaluated. PEEP was titrated according to oxygenation (PEEP_ARDSNetwork_), the lowest static elastance of the respiratory system (*E*_stat,RS_) [8] aiming to achieve the lowest *P*_driv_, thus determining a compromise between recruitment and overinflation [9, 10], and targeting a positive Ptp_exp_ to account for variability in lung and chest wall mechanics and optimize alveolar recruitment [11].

We found that (A) Ptp_exp_ and Ptp_insp_ increased using PEEP_ARDSNetwork_ and PEEP_Estat,RS_ during prone positioning; (B) PEEP and *P*_plat_ decreased when PEEP was titrated according to *E*_stat,RS_ or Ptp_exp_ in the prone position; (C) mechanical power was higher when PEEP was set using PEEP_Estat,RS_ and PEEP_Ptpexp_ strategies compared to PEEP_ARDSNetwork_ during supine positioning, and prone positioning attenuated this effect. In short, the PEEP titration strategy as well as the positioning significantly affected oxygenation, ventilatory and mechanical variables, as well as hemodynamics in patients with moderate to severe ARDS. PEEP_ARDSNetwork_ minimizes parameters associated with VILI while providing adequate gas exchange and preserves hemodynamics in both supine and prone positioning.

### Effects of prone positioning on transpulmonary pressure

Prone positioning induces substantial changes in lung mechanics because it reduces the compressive force of the mediastinum on the dependent lung regions, reduces pleural pressure [29], and thus modifies the vertical pleural pressure gradient [15]. From experimental [15, 30] and clinical [31, 32] studies, prone positioning promotes a more homogeneous distribution of regional aeration and compliance between the non-dependent and dependent lung compared with supine positioning. Riad et al. found an increase in the static elastance of the chest wall (*E*_stat,CW_) with prone positioning [32], which is in line with the results of this study as well as previous studies [33, 34]. The *E*_stat,CW_ may be further affected by increased IAP in the prone position [32–35]. Because *E*_stat,CW_ is increased in the prone position, ventilation and the resulting transpulmonary pressures are distributed more homogenously [36]. This has been shown to induce recruitment by shifting lung aeration more dorsally in patients with ARDS [37] and is associated with an improvement in gas exchange, ventilation/perfusion matching, and reduced shunting [33, 38, 39].

In our study, Ptp_exp_ and Ptp_insp_ increased in prone positioning compared to the supine position in PEEP_ARDSNetwork_ and PEEP_Estat,RS_ (Fig. 2) because of the decreased Pes_insp_ and Pes_exp_ (Table 1). Correspondingly Ptp_driv_ decreased in PEEP_ARDSNetwork_ following prone positioning (Fig. 4B) while *P*_driv_ remained unchanged. *P*_driv_ has been correlated with mortality in patients with ARDS [40], but it does not reflect the transmural pressure applied to the lung given the effect of prone positioning on chest wall mechanics [41]. Therefore, Ptp_driv_ might be the most important variable evaluated during PEEP titration in prone positioning as it represents true lung stress independent of chest wall mechanics [42].

### Effects of the PEEP strategy in supine and prone positioning

Setting PEEP to meet oxygenation goals (PEEP_ARDSNetwork_) resulted in the least invasive ventilator settings (i.e., *P*_plat_, PEEP, transpulmonary pressures, and mechanical power) (Fig. 3 and Table 1) and provided sufficient gas exchange and hemodynamics (Table 2). On the other hand, in the supine position, mechanical power due to *P*_plat_, PEEP, and transpulmonary pressures progressively increased with PEEP_Estat,RS_ and PEEP_Ptpexp_ (Table 1). The resulting *P*_plat_ increased similarly and was significantly higher for PEEP_Ptpexp_ (Fig. 3B). PEEP titration according to *E*_stat,RS_ and Ptp_exp_ has been evaluated in different clinical trials. The ART trial including patients with moderate to severe ARDS found increased mortality in patients randomized to PEEP_Estat,RS_ compared with PEEP_ARDSNetwork_. The investigators proposed breath stacking and dynamic overinflation as causative mechanisms [43]. Beitler et al. reported that a PEEP strategy based on Ptp_exp_ compared with PEEP_ARDSNetwork_ was not associated with better survival in patients with moderate to severe ARDS [23]. In a post hoc analysis, PEEP titrated to a Ptp_exp_ closer to 0 cm H_2_O was associated with greater survival than more positive or negative values, implying a reduction in alveolar cycling and hyperinflation [44]. Chiumello et al. evaluating different PEEP titration strategies found that the PEEP_ARDSNetwork_ was the only strategy where PEEP correlated with recruitability [45]. A recent meta-analysis found no beneficial effects on outcome when PEEP was set based on oxygenation or lowest *P*_driv_ [46]. In PEEP_ARDSNetwork_, Ptp_insp_ and Ptp_exp_ was higher with prone positioning (Fig. 2), presumably because of a corresponding decrease in esophageal (pleural) pressure, decreased Ptp_driv_ (Fig. 4B), and unchanged *P*_plat_, respiratory system, and lung elastance. The increase in EELV is associated with greater parenchymal aeration due to lung recruitment but not overdistension [19]. As highlighted by Gattinoni et al., the homogeneous distribution of transpulmonary pressure with dorsal shift of ventilation following prone positioning may improve recruitment while limiting alveolar overdistension [47]. Therefore, in the current study, the higher transpulmonary pressures during prone positioning might be associated with a favorable shift of ventilation and not overdistension. In fact, when using PEEP_ARDSNetwork_, EELV increased while *P*_driv_ and *P*_plat_ remained unchanged and Ptp_driv_ and mechanical power decreased after prone positioning. This finding of lung recruitment due to prone positioning with a low PEEP strategy is in line with the experimental findings from Scaramuzzo et al. [30] who found a significant recruitment of pulmonary parenchyma utilizing computed tomography and electrical impedance tomography. On the other hand, and albeit with significantly lower PEEP and *P*_plat_ in the prone position with the PEEP_Estat,RS_ strategy, *P*_driv_ and Ptp_driv_ did not change (Fig. 4A, B). *E*_stat,RS_ increased mainly due to an increased *E*_stat,CW_ and increased IAP with no further increase in EELV (Table 1). These findings are in line with the results of the experimental study by Katira et al. [15]. Because prone positioning reduced the vertical pleural pressure gradient, the effect of PEEP differs significantly from supine positioning. Lung homogeneity was maintained during a greater range of PEEP, and the level of PEEP to optimize the elastance of the dependent and non-dependent lung was lower compared with supine positioning. There is a ventral-dorsal pressure gradient of up to 10 cm H_2_O in the pleural space, therefore titrating PEEP to Ptp_exp_ optimizes end-expiratory aeration in the zone between the non-dependent and dependent lung regions [48]. In our study, the PEEP_Ptpexp_ strategy resulted in ventilator settings with the highest airway pressures applied and transferred the most energy in the lung in the supine as well as the prone position (Table 1). This is in line with the findings of Beitler et al. who also found a trend to higher *P*_plat_ and PEEP levels in the PEEP_Ptpexp_ group of patients in the EPVent-2 trial [23]. Notably, EELV was higher compared to PEEP_ARDSNetwork_ and PEEP_Estat,RS_ in the supine position but only compared with PEEP_ARDSNetwork_ in the prone position (Table 1).

### Effect of prone positioning on gas exchange and hemodynamics

PaO_2_/FiO_2_ differed between PEEP titration strategies with supine positioning but not prone positioning (Table 2). Prone positioning increased PaO_2_/FiO_2_ irrespective of the PEEP titration strategy. Prone positioning has been shown to improve gas exchange by homogenization of the gas/tissue ratio [49] and shape matching of the lungs and chest wall [33, 47]. Correspondingly, we found an increase in Ptp_exp_, Ptp_insp_, and *E*_stat,CW_ in the prone position, which potentially changed regional ventilation, improved ventilation/perfusion matching, and thus increased oxygenation and decreased dead space ventilation (Table 2). Furthermore, prone positioning significantly increased CI and MAP when using PEEP_Estat,RS_ and PEEP_Ptpexp_ (Table 2). Prone positioning has been shown to improve hemodynamics by several mechanisms. In preload-dependent patients, the increased IAP may improve venous return and thus cardiac output [50]. Right ventricular unloading is another beneficial effect of prone positioning because the improved gas exchange may limit hypoxic pulmonary vasoconstriction and permit a protective ventilation strategy with lower airway pressures [50]. The higher PEEP and *P*_plat_ when using PEEP_Estat,RS_ and PEEP_Ptpexp_ likely negatively affected cardiac pre- and afterload. This was presumably mitigated by the reduced airway pressures and increased IAP in the prone position.

### Clinical implications

Our data suggest that prone positioning increases Ptp_exp_ irrespective of the chosen PEEP strategy and thus permits a reduction in PEEP compared with supine positioning, minimizing airway pressures and mechanical power as well as improving oxygenation and hemodynamics. The use of a minimal PEEP level to achieve adequate oxygenation (PEEP_ARDSNetwork_) results in the most pronounced relative increase in Ptp_exp_ in the prone position compared with the other PEEP titration strategies. PEEP_ARDSNetwork_ caused the least invasive ventilator settings (i.e., Ptp_driv_ and mechanical power) without an increase in *P*_driv_ in prone positioning and with sufficient oxygenation and hemodynamics. This approach following the concept of "permissive atelectasis" might be sufficient to further minimize lung injury and VILI in lung protective ventilation [6].

### Limitations

Our study has several limitations that should be addressed. The non-randomized sequence of positioning and PEEP titration strategies may influence the results due to the longitudinal design with repeated measurements. To minimize a potential interaction between different PEEP titration strategies and positioning, a 30-min equilibration period between the measurements was permitted [51]. Similarly, a recruitment maneuver was performed to standardize the history of lung volume [52] between each measurement, although frequent recruitment maneuvers are not systematically recommended [5]. Individual recruitability was not assessed before the study to account for differences in lung morphology. This may have contributed to the limited recruitment effect of PEEP_Estat,RS_ and PEEP_Ptpexp_.

## Conclusions

Prone positioning increased transpulmonary pressures while improving oxygenation and hemodynamics in patients with moderate to severe ARDS when PEEP was titrated according to the ARDS Network lower PEEP table. This PEEP titration strategy minimized known parameters associated with VILI induction like transpulmonary driving pressure and mechanical power. We propose that a lower PEEP strategy (PEEP_ARDSNetwork_) in combination with prone positioning may be part of a lung protective ventilation strategy in patients with moderate to severe ARDS.

## Supplementary Information

**Additional file 1.** Study details, calculations, and additional analysis of the effects of three different PEEP titration strategies during supine and prone positioning.

## Data Availability

The datasets analyzed during this study are available from the corresponding author on reasonable request.

## Abbreviations

ARDS: Acute respiratory distress syndrome
CI: Cardiac index
CVP: Central venous pressure
EELV: End-expiratory lung volume
*E*_stat,RS_: Static elastance of the respiratory system
*E*_stat,CW_: Static elastance of the chest wall
FiO_2_: Fraction of inspired oxygen
IAP: Intraabdominal pressure
ICU: Intensive care unit
MAP: Mean arterial pressure
PaO_2_/FiO_2_: Ratio of arterial oxygen partial pressure divided by the fraction of inspired oxygen
*P*_driv_: Driving pressure
PEEP: Positive end-expiratory pressure
PEEP_ARDSNetwork_: PEEP titrated according to the ARDS Network lower PEEP table
PEEP_Estat,RS_: PEEP titrated according to the lowest static elastance of the respiratory system
PEEP_Ptpexp_: PEEP titrated according to the end-expiratory transpulmonary pressure
Pes_exp_: End-expiratory esophageal pressure
Pes_insp_: End-inspiratory esophageal pressure
pHa: Negative logarithm of the molar concentration of dissolved hydronium ions in arterial blood
*P*_plat_: End-inspiratory plateau airway pressure
Ptp_driv_: Transpulmonary driving pressure
Ptp_exp_: End-expiratory transpulmonary pressure
Ptp_insp_: End-inspiratory transpulmonary pressure
RR: Respiratory rate
SAPS II: Simplified Acute Physiology Score II
S_cv_O_2_: Central venous oxygen saturation
SOFA: Sequential Organ Failure Assessment
*V*_D_/*V*_T_: Ratio of physiologic dead space to tidal volume
VILI: Ventilator-induced lung injury
*V*_T_: Tidal volume
