# Supplementary material for: Effects of different positive end-expiratory pressure titration strategies during prone positioning in patients with acute respiratory distress syndrome: a prospective interventional study
Source: Crit Care. 2022 Mar 26;26:82. doi: 10.1186/s13054-022-03956-8 (PMC8962042; doi:10.1186/s13054-022-03956-8)
Supplement: Supplementary file 1 — Additional file 1. Study details, calculations, and additional analysis of the effects of three different PEEP titration strategies during supine and prone positioning. [file 13054_2022_3956_MOESM1_ESM.docx]

Effects of Different Positive End-Expiratory Pressure Titration Strategies During Prone Positioning in Patients with Acute Respiratory Distress Syndrome: a Prospective Interventional Study

Additional file 1

Christoph Boesing^1^; Peter T. Graf^1^; Fabian Schmitt^1^; Manfred Thiel^1^; Paolo Pelosi^2,3^; Patricia RM Rocco^4^; Thomas Luecke^1^; Joerg Krebs^1^

^1^Department of Anaesthesiology and Critical Care Medicine, University Medical Centre Mannheim, Medical Faculty Mannheim of the University of Heidelberg, Theodor-Kutzer Ufer 1-3, 68167 Mannheim, Germany

^2^Department of Surgical Sciences and Integrated Diagnostics, University of Genoa, Genoa, Italy

^3^Anesthesiology and Critical Care - San Martino Policlinico Hospital, IRCCS for Oncology and Neurosciences, Genoa, Italy

^4^Laboratory of Pulmonary Investigation, Carlos Chagas Filho Institute of Biophysics, Federal University of Rio de Janeiro, Centro de Ciências da Saúde, Avenida Carlos Chagas Filho, 373, Bloco G-014, Ilha do Fundão, Rio de Janeiro, Brazil

**E-mail addresses:**

Christoph Boesing: christoph.boesing@umm.de

Peter T. Graf: tobias.graf@umm.de

Fabian Schmitt: fabian.schmitt@medma.uni-heidelberg.de

Manfred Thiel: manfred.thiel@umm.de

Paolo Pelosi: ppelosi@hotmail.com

Patricia RM Rocco: prmrocco@biof.ufrj.br

Thomas Luecke: thomas.luecke@medma.uni-heidelberg.de

**Corresponding author:**

Joerg Krebs, Department of Anaesthesiology and Critical Care Medicine, University Medical Centre Mannheim, Theodor-Kutzer-Ufer 1-3, 68167 Mannheim. joerg.krebs@umm.de

**Table S1.** Allowable combinations of fraction of inspired oxygen and PEEP

| **Allowable combinations of PEEP and FiO_2_** | | | | | | | | | | | | | | |
| --- | --- | --- | --- | --- | --- | --- | --- | --- | --- | --- | --- | --- | --- | --- |
| **FiO_2_ (%)** | 30 | 40 | 40 | 50 | 50 | 60 | 70 | 70 | 70 | 80 | 90 | 90 | 90 | 100 |
| **PEEP (cm H_2_O)** | 5 | 5 | 8 | 8 | 10 | 10 | 10 | 12 | 14 | 14 | 14 | 16 | 18 | 18–24 |

PEEP and FiO_2_ were titrated to achieve an arterial oxygen saturation of 88%–92%.

Adapted from (1).

*FiO_2_* fraction of inspired oxygen, *PEEP* positive end-expiratory pressure

**Figure S1.** Schematic representation of the dynamic recruitment maneuver and the decremental PEEP trial


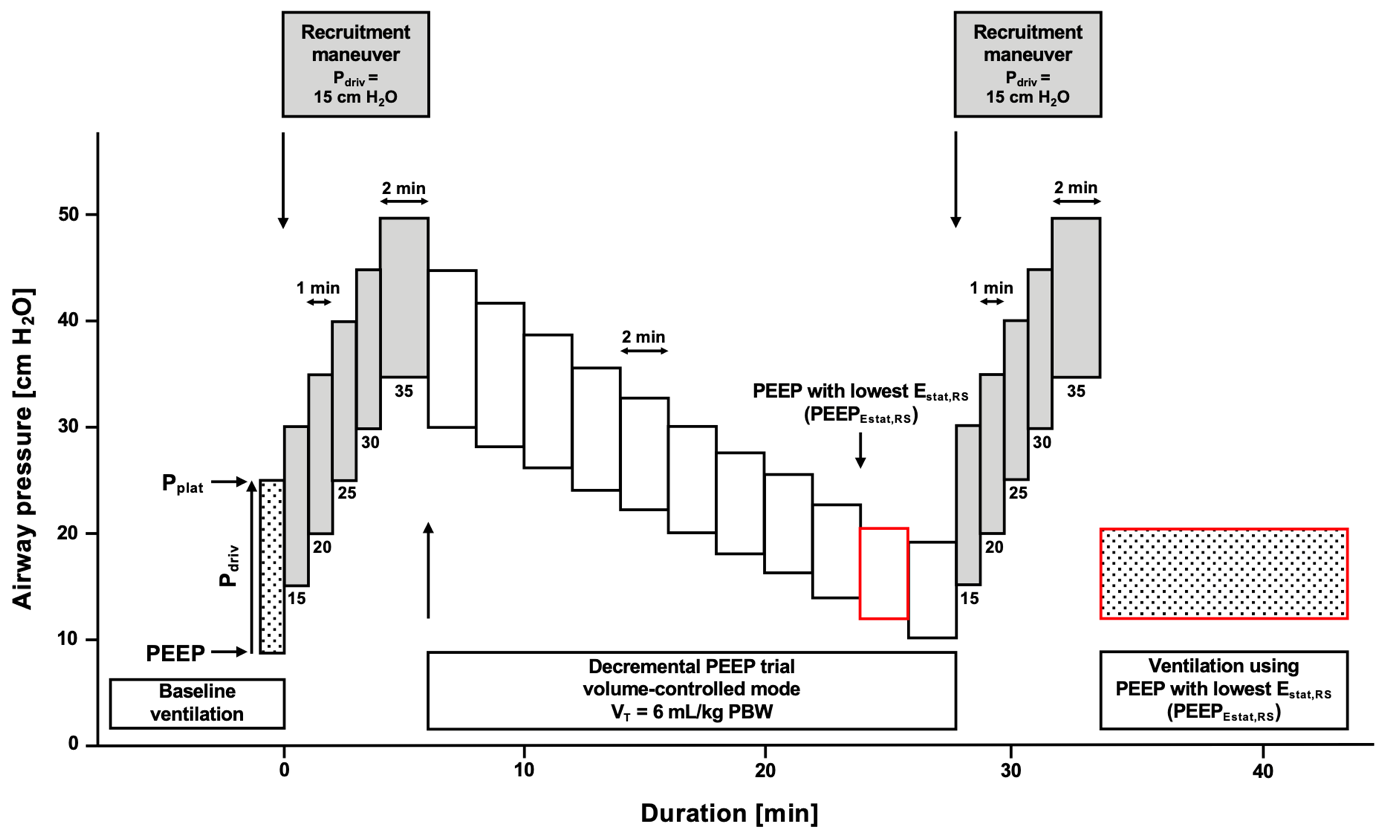


*E_stat,RS_* static elastance of the respiratory system, *PBW* predicted body weight, *P_driv_* driving pressure, *PEEP* positive end-expiratory pressure, *PEEP_Estat,RS_* PEEP titrated according to the lowest elastance of the respiratory system, *P_plat_* end-inspiratory plateau pressure, *V_T_* tidal volume

PEEP was slowly increased to 35 cm H_2_O in a pressure-controlled mode with a P_driv_ of 15 cm H_2_O over a period of 5 minutes starting with a PEEP of 15 cm H_2_O. After 2 minutes with a PEEP of 35 cm H_2_O, ventilator mode was switched back to a volume-controlled mode using the initial tidal volume and respiratory rate to perform a decremental PEEP trial. Starting with a PEEP of 30 cm H_2_O, the PEEP was reduced in steps of 2 cm H_2_O every 2 minutes. PEEP was considered optimal at the step with the lowest E_stat,RS_ (PEEP_Estat,RS_), and then another dynamic recruitment maneuver was performed..

**Table S2.** Allowable combinations of fraction of inspired oxygen and end-expiratory transpulmonary pressures

| **Allowable combinations of FiO_2_ and Ptp_exp_** | | | | | | | | | | | | | |
| --- | --- | --- | --- | --- | --- | --- | --- | --- | --- | --- | --- | --- | --- |
| **FiO_2_ (%)** | 30 | 40 | 50 | 50 | 60 | 60 | 70 | 70 | 80 | 80 | 90 | 90 | 100 |
| **Ptpexp (cm H_2_O)** | 0 | 0 | 0 | 2 | 2 | 3 | 3 | 4 | 4 | 5 | 5 | 6 | 6 |

PEEP and Ptp_exp_ were titrated to achieve an arterial oxygen saturation of 88%–92%.

Adapted from (2).

*FiO_2_* fraction of inspired oxygen, *PEEP* positive end-expiratory pressure, *Ptp_exp_* end-expiratory transpulmonary pressure

## Calculations for Respiratory Parameters

Driving pressure (P_driv_) = end-inspiratory plateau airway pressure (P_plat_) − PEEP

End-inspiratory transpulmonary pressure (Ptp_insp_) = P_plat_ − end-inspiratory esophageal pressure (Pes_insp_)

End-expiratory transpulmonary pressure (Ptp_exp_) = PEEP − end-expiratory esophageal pressure (Pes_exp_)

Difference between esophageal pressure at P_plat_ and PEEP (∆Pes) = Pes_insp_ − Pes_exp_

Static elastance of the respiratory system (E_stat,RS_) = (P_plat_ − PEEP)/tidal volume (V_T_)

Static elastance of the chest wall (E_stat,CW_) = ∆Pes/V_T_

Static elastance of the lung (E_stat,L_) = E_stat,RS_ − E_stat,CW_

Mechanical power = 0.098 × V_T_ × Respiratory rate × (Peak inspiratory pressure − P_driv_/2)
 Calculation based on the simplified formula for volume-controlled ventilation according to (3)

Ratio of physiologic dead space to tidal volume (V_D_/V_T_) = (arterial partial pressure of carbon dioxide (PaCO_2_) − mean partial pressure of exhaled CO_2_)/PaCO_2_

Ventilatory ratio = (Respiratory rate × V_T_ × PaCO_2_)/(100 mL/min/kg body weight × 37)
 Calculation based on the formula according to (4)

**Table S3.** Demographic and clinical characteristics of the patients included in the study

| **Characteristics** | ***N* = 40** |
| --- | --- |
| **Age (years)** | 63.7 ± 16.2 |
| **Male sex (%)** | 70 |
| **Body mass index (kg/m^2^)** | 29.8 ± 6.6 |
| **MV before study (days)** | 5.6 ± 4.7 |
| **Cause of ARDS** |  |
| **Pulmonary bacterial (*n*)** | 10 |
| **Pulmonary viral (*n*)** | 22 |
| **Extrapulmonary (*n*)** | 8 |
| **SAPS II** | 71.7 ± 12.5 |
| **SOFA** | 10.5 ± 2.4 |
| **Length of stay ICU (days)** | 19.9 ± 13.6 |
| **ICU mortality (%)** | 37.5 |

Data from 40 patients with moderate to severe ARDS are reported as means ± standard deviation or percentages as appropriate.

*ICU* intensive care unit, *MV* mechanical ventilation, *SAPS II* Simplified Acute Physiology Score II, *SOFA* Sequential Organ Failure Assessment

**Table S4.** Mechanics of the respiratory system, mechanical power, and end-expiratory lung volume for three PEEP titration strategies during supine positioning

|  | **Supine** | | | ***p* value** | ***p* value** | ***p* value** |
| --- | --- | --- | --- | --- | --- | --- |
|  | **PEEP_ARDSNetwork_** | **PEEP_Estat,RS_** | **PEEP_Ptpexp_** | **PEEP_ARDSNetwork_ vs. PEEP_Estat,RS_** | **PEEP_ARDSNetwork_ vs. PEEP_Ptpexp_** | **PEEP_Estat,RS_ vs. PEEP_Ptpexp_** |
| **RR (breaths/min)** | 22.3 ± 1.9 | 22.3 ± 1.9 | 22.3 ± 1.9 | 1.000 | 1.000 | 1.000 |
| **V_T_ (mL/kg PBW)** | 6.2 ± 0.3 | 6.2 ± 0.3 | 6.2 ± 0.3 | 1.000 | 1.000 | 1.000 |
| **P_peak,RS_ (cm H_2_O)** | 23.3 ± 5.1 | 25.0 ± 5.1 | 30.6 ± 8.8 | 0.085 | **<0.001** | **<0.001** |
| **P_plat_ (cm H_2_O)** | 17.5 ± 3.7 | 19.5 ± 4.2 | 24.5 ± 7.1 | **0.029** | **<0.001** | **<0.001** |
| **PEEP (cm H_2_O)** | 8.9 ± 2.6 | 11.6 ± 3.9 | 16.1 ± 5.8 | **<0.001** | **<0.001** | **<0.001** |
| **P_mean,RS_ (cm H_2_O)** | 13.5 ± 2.9 | 15.8 ± 4.0 | 20.5 ± 6.1 | **0.002** | **<0.001** | **<0.001** |
| **P_driv_ (cm H_2_O)** | 8.6 ± 2.3 | 7.9 ± 1.8 | 8.4 ± 2.8 | **0.046** | 0.143 | 0.512 |
| **Pes_insp_ (cm H_2_O)** | 14.2 ± 3.5 | 14.8 ± 4.4 | 17.1 ± 5.7 | 0.488 | **<0.001** | **<0.001** |
| **Pes_exp_ (cm H_2_O)** | 11.3 ± 3.1 | 12.1 ± 4.0 | 13.4 ± 5.4 | 0.154 | **0.002** | 0.070 |
| **∆Pes (cm H_2_O)** | 2.9 ± 1.6 | 2.7 ± 1.5 | 3.0 ± 1.8 | 0.149 | 0.149 | 0.149 |
| **Ptp_insp_ (cm H_2_O)** | 3.3 ± 4.2 | 4.7 ± 3.0 | 7.5 ± 4.1 | **0.009** | **<0.001** | **<0.001** |
| **Ptp_exp_ (cm H_2_O)** | −2.4 ± 3.5 | −0.5 ± 2.1 | 2.0 ± 2.3 | **<0.001** | **<0.001** | **<0.001** |
| **Ptp_driv_ (cm H_2_O)** | 5.7 ± 2.2 | 5.2 ± 1.9 | 5.5 ± 2.7 | 0.278 | 0.614 | 0.876 |
| **E_stat,RS_ (cm H_2_O/L)** | 21.3 ± 6.5 | 19.5 ± 5.8 | 20.7 ± 8.0 | 0.059 | 0.059 | 0.059 |
| **E_stat,CW_ (cm H_2_O/L)** | 7.1 ± 4.2 | 6.6 ± 4.2 | 7.3 ± 4.8 | 0.165 | 0.165 | 0.165 |
| **E_stat,L_ (cm H_2_O/L)** | 14.0 ± 5.5 | 12.8 ± 4.8 | 13.4 ± 6.9 | 0.179 | 0.179 | 0.179 |
| **Mechanical power (J/min)** | 17.5 ± 5.7 | 19.2 ± 5.9 | 24.4 ± 9.8 | 0.054 | **<0.001** | **<0.001** |
| **IAP (cm H_2_O)** | 8.3 ± 2.8 | 8.7 ± 2.9 | 9.9 ± 3.5 | 0.093 | **<0.001** | **<0.001** |
| **V_D_/V_T_ (%)** | 28.5 ± 10.9 | 27.6 ± 10.8 | 28.4 ± 10.5 | 0.506 | 0.506 | 0.506 |
| **Ventilatory rate** | 2.09 ± 0.5 | 2.12 ± 0.5 | 2.13 ± 0.5 | 0.844 | 0.841 | 0.992 |
| **EELV (mL)** | 1630 ± 552 | 1920 ± 556 | 2140 ± 615 | **<0.001** | **<0.001** | 0.052 |

Values are means ± standard deviation of 40 patients with moderate to severe ARDS. Repeated measures ANOVA followed by Holm-Sidak’s post hoc test was used to compare the effects of different PEEP titration strategies on respiratory parameters during supine positioning (*p* < 0.05).

*∆Pes* difference between esophageal pressure at plateau airway pressure and positive end-expiratory pressure, *EELV* end-expiratory lung volume, *E_stat,CW_* static elastance of the chest wall, *E_stat,L_* static elastance of the lung, *E_stat,RS_* static elastance of the respiratory system, *IAP* intraabdominal pressure, *PBW* predicted body weight, *P_driv_* driving pressure, *PEEP* positive end-expiratory pressure, *PEEP_ARDSNetwork_* PEEP titrated according to the ARDS Network lower PEEP table, *PEEP_Estat,RS_* PEEP titrated according to the lowest elastance of the respiratory system, *PEEP_Ptpexp_* PEEP titrated according to end-expiratory transpulmonary pressure, *Pes_exp_* esophageal pressure at positive end-expiratory pressure, *Pes_insp_* esophageal pressure at plateau airway pressure, *P_mean,RS_* mean airway pressure of the respiratory system, *P_peak,RS_* peak airway pressure of the respiratory system, *P_plat_* end-inspiratory plateau airway pressure, *Ptp_driv_* transpulmonary driving pressure, *Ptp_exp_* end-expiratory transpulmonary pressure, *Ptp_insp_* end-inspiratory transpulmonary pressure, *RR* respiratory rate, *V_D_/V_T_* ratio of physiologic dead space to tidal volume, *V_T_* tidal volume

**Table S5.** Mechanics of the respiratory system, mechanical power, and end-expiratory lung volume for three PEEP titration strategies during prone positioning

|  | **Prone** | | | ***p* value** | ***p* value** | ***p* value** |
| --- | --- | --- | --- | --- | --- | --- |
|  | **PEEP_ARDSNetwork_** | **PEEP_Estat,RS_** | **PEEP_Ptpexp_** | **PEEP_ARDSNetwork_ vs. PEEP_Estat,RS_** | **PEEP_ARDSNetwork_ vs. PEEP_Ptpexp_** | **PEEP_Estat,RS_ vs. PEEP_Ptpexp_** |
| **RR (breaths/min)** | 22.3 ± 1.9 | 22.3 ± 1.9 | 22.3 ± 1.9 | 1.000 | 1.000 | 1.000 |
| **V_T_ (mL/kg PBW)** | 6.2 ± 0.3 | 6.2 ± 0.3 | 6.2 ± 0.3 | 1.000 | 1.000 | 1.000 |
| **P_peak,RS_ (cm H_2_O)** | 23.5 ± 4.5 | 23.3 ± 4.5 | 27.2 ±8.8 | 0.824 | **<0.001** | **<0.001** |
| **P_plat_ (cm H_2_O)** | 17.6 ± 3.4 | 17.5 ± 3.3 | 20.9 ± 7.2 | 0.871 | **0.001** | **0.001** |
| **PEEP (cm H_2_O)** | 8.8 ± 2.6 | 9.0 ± 3.3 | 11.8 ± 6.3 | **0.001** | 0.245 | **0.002** |
| **P_mean,RS_ (cm H_2_O)** | 13.5 ± 2.7 | 13.6 ± 3.4 | 16.5 ± 6.5 | 0.854 | **<0.001** | **<0.001** |
| **P_driv_ (cm H_2_O)** | 8.9 ± 2.0 | 8.5 ± 1.7 | 9.1 ± 2.4 | 0.338 | 0.182 | **0.029** |
| **Pes_insp_ (cm H_2_O)** | 11.4 ± 3.7 | 11.4 ± 4.6 | 13.0 ± 6.0 | 0.917 | **0.002** | **0.002** |
| **Pes_exp_ (cm H_2_O)** | 7.7 ± 3.2 | 7.9 ± 4.0 | 9.4 ± 5.4 | 0.671 | **<0.001** | **<0.001** |
| **∆Pes (cm H_2_O)** | 3.6 ± 1.9 | 3.5 ± 1.9 | 3.5 ± 2.0 | 0.410 | 0.410 | 0.410 |
| **Ptp_insp_ (cm H_2_O)** | 6.3 ± 4.6 | 6.1 ± 3.5 | 8.0 ± 4.8 | 0.757 | **0.009** | **0.005** |
| **Ptp_exp_ (cm H_2_O)** | 1.1 ± 3.4 | 1.1 ± 2.5 | 2.4 ± 2.7 | 0.957 | **0.014** | **0.011** |
| **Ptp_driv_ (cm H_2_O)** | 5.2 ± 2.1 | 5.0 ± 2.2 | 5.5 ± 2.9 | 0.684 | 0.603 | 0.394 |
| **E_stat,RS_ (cm H_2_O/L)** | 21.7 ± 6.0 | 20.8 ± 5.2 | 22.1 ± 6.4 | 0.143 | 0.346 | **0.022** |
| **E_stat,CW_ (cm H_2_O/L)** | 9.0 ± 5.0 | 8.6 ± 4.9 | 8.8 ± 5.2 | 0.403 | 0.403 | 0.403 |
| **E_stat,L_ (cm H_2_O/L)** | 13.0 ± 5.3 | 12.6 ± 5.4 | 13.9 ± 6.8 | 0.314 | 0.176 | **0.024** |
| **Mechanical power (J/min)** | 17.6 ± 5.5 | 17.5 ± 5.8 | 21.0 ± 9.6 | 0.931 | **<0.001** | **0.001** |
| **IAP (cm H_2_O)** | 11.1 ± 3.0 | 11.1 ± 3.5 | 12.1 ± 3.8 | 0.941 | **0.007** | **0.009** |
| **V_D_/V_T_ (%)** | 25.2 ± 9.0 | 24.7 ± 9.5 | 25.8 ± 9.5 | 0.193 | 0.193 | 0.193 |
| **Ventilatory rate** | 2.11 ± 0.5 | 2.13 ± 0.5 | 2.14 ± 0.5 | 0.570 | 0.421 | 0.226 |
| **EELV (mL)** | 1972 ± 693 | 1993 ± 627 | 2108 ± 736 | 0.804 | **0.027** | **0.035** |

Values are means ± standard deviation of 40 patients with moderate to severe ARDS. Repeated measures ANOVA followed by Holm-Sidak’s post hoc test was used to compare the effects of different PEEP titration strategies on respiratory parameters during prone positioning (*p* < 0.05).

*∆Pes* difference between esophageal pressure at plateau airway pressure and positive end-expiratory pressure, *EELV* end-expiratory lung volume, *E_stat,CW_* static elastance of the chest wall, *E_stat,L_* static elastance of the lung, *E_stat,RS_* static elastance of the respiratory system, *IAP* intraabdominal pressure, *PBW* predicted body weight, *P_driv_* driving pressure, *PEEP* positive end-expiratory pressure, *PEEP_ARDSNetwork_* PEEP titrated according to the ARDS Network lower PEEP table, *PEEP_Estat,RS_* PEEP titrated according to the lowest elastance of the respiratory system, *PEEP_Ptpexp_* PEEP titrated according to end-expiratory transpulmonary pressure, *Pes_exp_* esophageal pressure at positive end-expiratory pressure, *Pes_insp_* esophageal pressure at plateau airway pressure, *P_mean,RS_* mean airway pressure of the respiratory system, *P_peak,RS_* peak airway pressure of the respiratory system, *P_plat_* end-inspiratory plateau airway pressure, *Ptp_driv_* transpulmonary driving pressure, *Ptp_exp_* end-expiratory transpulmonary pressure, *Ptp_insp_* end-inspiratory transpulmonary pressure, *RR* respiratory rate, *V_D_/V_T_* ratio of physiologic dead space to tidal volume, *V_T_* tidal volume

**Table S6.** Gas exchange and hemodynamics of three PEEP titration strategies during supine positioning

|  | **Supine** | | | ***p* value** | ***p* value** | ***p* value** | |
| --- | --- | --- | --- | --- | --- | --- | --- |
|  | **PEEP_ARDSNetwork_** | **PEEP_Estat,RS_** | **PEEP_Ptpexp_** | **PEEP_ARDSNetwork_ vs. PEEP_Estat,RS_** | **PEEP_ARDSNetwork_ vs. PEEP_Ptpexp_** | | **PEEP_Estat,RS_ vs. PEEP_Ptpexp_** |
| **PaO_2_/FiO_2_ (mm Hg)** | 136 ± 36 | 170 ± 72 | 192 ± 76 | **0.001** | **<0.001** | **0.026** | |
| **PaCO_2_ (mm Hg)** | 57.0 ± 10.5 | 57.6 ± 10.3 | 57.6 ± 10.4 | 0.246 | 0.246 | 0.246 | |
| **pHa** | 7.3 ± 0.1 | 7.3 ± 0.1 | 7.3 ± 0.1 | 0.055 | 0.055 | 0.055 | |
| **HR (beats/min)** | 92.6 ± 19.1 | 92.8 ± 20.5 | 92.6 ± 19.2 | 0.925 | 0.925 | 0.925 | |
| **MAP (mm Hg)** | 83.0 ± 10.8 | 82.6 ± 9.8 b | 79.2 ± 11.3 | 0.737 | **0.003** | **0.006** | |
| **Noradrenaline (µg/kg/min)** | 0.2 ± 0.3 | 0.2 ± 0.3 | 0.2 ± 0.2 | 0.584 | 0.584 | 0.584 | |
| **CVP (mm Hg)** | 14.1 ± 6.4 | 15.1 ± 6.9 | 16.4 ± 7.4 | **0.005** | **0.001** | **<0.001** | |
| **S_cv_O_2_ (%)** | 75.3 ± 7.4 | 77.6 ± 5.2 | 77.6 ± 6.1 | 0.141 | 0.128 | 0.998 | |
| **CI (L/min/m^2^)** | 3.7 ± 1.0 | 3.5 ± 0.9 b | 3.2 ± 0.7 | **0.048** | **<0.001** | **<0.001** | |

Values are means ± standard deviation of 40 patients with moderate to severe ARDS. Repeated measures ANOVA followed by Holm-Sidak’s post hoc test was used to compare the effects of different PEEP titration strategies on gas exchange and hemodynamics during supine positioning (*p* < 0.05).

*CI* cardiac index, *CVP* central venous pressure, *HR* heart rate, *MAP* mean arterial pressure, *PaCO_2_* arterial partial pressure of carbon dioxide, *PEEP* positive end-expiratory pressure, *PEEP_ARDSNetwork_* PEEP titrated according to the ARDS Network lower PEEP table, *PEEP_Estat,RS_* PEEP titrated according to the lowest elastance of the respiratory system, *PEEP_Ptpexp_* PEEP titrated according to end-expiratory transpulmonary pressure, *PaO_2_/FiO_2_* arterial oxygen partial pressure divided by the fraction of inspired oxygen, *pHa* negative logarithm of the molar concentration of dissolved hydronium ions in arterial blood, *S_cv_O_2_* central venous oxygen saturation

**Table S7.** Gas exchange and hemodynamics of three PEEP titration strategies during prone positioning

|  | **Prone** | | | ***p* value** | ***p* value** | ***p* value** | |
| --- | --- | --- | --- | --- | --- | --- | --- |
|  | **PEEP_ARDSNetwork_** | **PEEP_Estat,RS_** | **PEEP_Ptpexp_** | **PEEP_ARDSNetwork_ vs. PEEP_Estat,RS_** | **PEEP_ARDSNetwork_ vs. PEEP_Ptpexp_** | | **PEEP_Estat,RS_ vs. PEEP_Ptpexp_** |
| **PaO_2_/FiO_2_ (mm Hg)** | 228 ± 86 | 237 ± 91 | 240 ± 100 | 0.285 | 0.285 | 0.285 | |
| **PaCO_2_ (mm Hg)** | 57.9 ± 10.5 | 57.7 ± 10.7 | 58.0 ± 10.4 | 0.470 | 0.470 | 0.470 | |
| **pHa** | 7.3 ± 0.1 | 7.3 ± 0.1 | 7.3 ± 0.1 | 0.113 | 0.113 | 0.113 | |
| **HR (beats/min)** | 93.1 ± 21.2 | 93.2 ± 21.4 | 92.4 ± 20.6 | 0.431 | 0.431 | 0.431 | |
| **MAP (mm Hg)** | 87.1 ± 11.2 | 89.5 ± 11.9 | 87.6 ± 11.4 | 0.077 | 0.077 | 0.077 | |
| **Noradrenaline (µg/kg/min)** | 0.2 ± 0.3 | 0.2 ± 0.3 | 0.2 ± 0.2 | 0.592 | 0.592 | 0.592 | |
| **CVP (mm Hg)** | 16.8 ± 5.6 | 16.8 ± 6.2 | 17.5 ± 6.8 | 0.052 | 0.052 | 0.052 | |
| **S_cv_O_2_ (%)** | 81.2 ± 6.8 | 82.8 ± 6.2 | 81.4 ± 6.3 | 0.279 | 0.921 | 0.309 | |
| **CI (L/min/m^2^)** | 3.7 ± 0.9 | 3.7 ± 1.0 | 3.6 ± 0.8 | 0.957 | **0.024** | **0.019** | |

Values are means ± standard deviation of 40 patients with moderate to severe ARDS. Repeated measures ANOVA followed by Holm-Sidak’s post hoc test was used to compare the effects of different PEEP titration strategies on gas exchange and hemodynamics during prone positioning (*p* < 0.05).

*CI* cardiac index, *CVP* central venous pressure, *HR* heart rate, *MAP* mean arterial pressure, *PaCO_2_* arterial partial pressure of carbon dioxide, *PEEP* positive end-expiratory pressure, *PEEP_ARDSNetwork_* PEEP titrated according to the ARDS Network lower PEEP table, *PEEP_Estat,RS_* PEEP titrated according to the lowest elastance of the respiratory system, *PEEP_Ptpexp_* PEEP titrated according to end-expiratory transpulmonary pressure, *PaO_2_/FiO_2_* arterial oxygen partial pressure divided by the fraction of inspired oxygen, *pHa* negative logarithm of the molar concentration of dissolved hydronium ions in arterial blood, *S_cv_O_2_* central venous oxygen saturation

**Table S8.** Mechanics of the respiratory system, mechanical power, and end-expiratory lung volume for three PEEP titration strategies during supine and prone positioning

|  | **PEEP_ARDSNetwork_** | |  | **PEEP_Estat,RS_** | |  | **PEEP_Ptpexp_** | |  |
| --- | --- | --- | --- | --- | --- | --- | --- | --- | --- |
|  | **Supine** | **Prone** | ***p* value** | **Supine** | **Prone** | ***p* value** | **Supine** | **Prone** | ***p* value** |
| **RR (breaths/min)** | 22.3 ± 1.9 | 22.3 ± 1.9 | 1.000 | 22.3 ± 1.9 | 22.3 ± 1.9 | 1.000 | 22.3 ± 1.9 | 22.3 ± 1.9 | 1.000 |
| **V_T_ (mL/kg PBW)** | 6.2 ± 0.3 | 6.2 ± 0.3 | 1.000 | 6.2 ± 0.3 | 6.2 ± 0.3 | 1.000 | 6.2 ± 0.3 | 6.2 ± 0.3 | 1.000 |
| **P_peak,RS_ (cm H_2_O)** | 23.3 ± 5.1 | 23.5 ± 4.5 | 0.642 | 25.0 ± 5.1 | 23.3 ± 4.5 | **0.005** | 30.6 ± 8.8 | 27.2 ±8.8 | **<0.001** |
| **P_plat,RS_ (cm H_2_O)** | 17.5 ± 3.7 | 17.6 ± 3.4 | 0.257 | 19.5 ± 4.2 | 17.5 ± 3.3 | **0.040** | 24.5 ± 7.1 | 20.9 ± 7.2 | **<0.001** |
| **PEEP (cm H_2_O)** | 8.9 ± 2.6 | 8.8 ± 2.6 | 0.781 | 11.6 ± 3.9 | 9.0 ± 3.3 | **<0.001** | 16.1 ± 5.8 | 11.8 ± 6.3 | **<0.001** |
| **P_mean,RS_ (cm H_2_O)** | 13.5 ± 2.9 | 13.5 ± 2.7 | 0.297 | 15.8 ± 4.0 | 13.6 ± 3.4 | **<0.001** | 20.5 ± 6.1 | 16.5 ± 6.5 | **<0.001** |
| **P_driv_ (cm H_2_O)** | 8.6 ± 2.3 | 8.9 ± 2.0 | 0.064 | 7.9 ± 1.8 | 8.5 ± 1.7 | **0.002** | 8.4 ± 2.8 | 9.1 ± 2.4 | **0.020** |
| **Pes_insp_ (cm H_2_O)** | 14.2 ± 3.5 | 11.4 ± 3.7 | **<0.001** | 14.8 ± 4.4 | 11.4 ± 4.6 | **<0.001** | 17.1 ± 5.7 | 13.0 ± 6.0 | **<0.001** |
| **Pes_exp_ (cm H_2_O)** | 11.3 ± 3.1 | 7.7 ± 3.2 | **<0.001** | 12.1 ± 4.0 | 7.9 ± 4.0 | **<0.001** | 13.4 ± 5.4 | 9.4 ± 5.4 | **<0.001** |
| **∆Pes (cm H_2_O)** | 2.9 ± 1.6 | 3.6 ± 1.9 | **<0.001** | 2.7 ± 1.5 | 3.5 ± 1.9 | **<0.001** | 3.0 ± 1.8 | 3.5 ± 2.0 | **0.009** |
| **Ptp_insp_ (cm H_2_O)** | 3.3 ± 4.2 | 6.3 ± 4.6 | **<0.001** | 4.7 ± 3.0 | 6.1 ± 3.5 | **0.002** | 7.5 ± 4.1 | 8.0 ± 4.8 | 0.597 |
| **Ptp_exp_ (cm H_2_O)** | −2.4 ± 3.5 | 1.1 ± 3.4 | **<0.001** | −0.5 ± 2.1 | 1.1 ± 2.5 | **<0.001** | 2.0 ± 2.3 | 2.4 ± 2.7 | 0.201 |
| **Ptp_driv_ (cm H_2_O)** | 5.7 ± 2.2 | 5.2 ± 2.1 | **0.031** | 5.2 ± 1.9 | 5.0 ± 2.2 | 0.312 | 5.5 ± 2.7 | 5.5 ± 2.9 | 0.643 |
| **E_stat,RS_ (cm H_2_O/L)** | 21.3 ± 6.5 | 21.7 ± 6.0 | 0.537 | 19.5 ± 5.8 | 20.8 ± 5.2 | **0.004** | 20.7 ± 8.0 | 22.1 ± 6.4 | **0.044** |
| **E_stat,CW_ (cm H_2_O/L)** | 7.1 ± 4.2 | 9.0 ± 5.0 | **<0.001** | 6.6 ± 4.2 | 8.6 ± 4.9 | **<0.001** | 7.3 ± 4.8 | 8.8 ± 5.2 | **0.003** |
| **E_stat,L_ (cm H_2_O/L)** | 14.0 ± 5.5 | 13.0 ± 5.3 | 0.081 | 12.8 ± 4.8 | 12.6 ± 5.4 | 0.621 | 13.4 ± 6.9 | 13.9 ± 6.8 | 0.515 |
| **Mechanical power (J/min)** | 17.5 ± 5.7 | 17.6 ± 5.5 | 0.743 | 19.2 ± 5.9 | 17.5 ± 5.8 | **<0.001** | 24.4 ± 9.8 | 21.0 ± 9.6 | **<0.001** |
| **IAP (cm H_2_O)** | 8.3 ± 2.8 | 11.1 ± 3.0 | **<0.001** | 8.7 ± 2.9 | 11.1 ± 3.5 | **<0.001** | 9.9 ± 3.5 | 12.1 ± 3.8 | **<0.001** |
| **V_D_/V_T_ (%)** | 28.5 ± 10.9 | 25.2 ± 9.0 | **0.002** | 27.6 ± 10.8 | 24.7 ± 9.5 | **0.011** | 28.4 ± 10.5 | 25.8 ± 9.5 | **0.003** |
| **Ventilatory rate** | 2.09 ± 0.5 | 2.11 ± 0.5 | 0.080 | 2.12 ± 0.5 | 2.13 ± 0.5 | 0.587 | 2.13 ± 0.5 | 2.14 ± 0.5 | 0.219 |
| **EELV (mL)** | 1630 ± 552 | 1972 ± 693 | **0.026** | 1920 ± 556 | 1993 ± 627 | 0.947 | 2140 ± 615 | 2108 ± 736 | 0.330 |

Values are means ± standard deviation of 40 patients with moderate to severe ARDS. Repeated measures ANOVA followed by Holm-Sidak’s post hoc test was used to compare the effects of different PEEP titration strategies on respiratory parameters during supine and prone positioning (*p* < 0.05).

*∆Pes* difference between esophageal pressure at plateau airway pressure and positive end-expiratory pressure, *EELV* end-expiratory lung volume, *E_stat,CW_* static elastance of the chest wall, *E_stat,L_* static elastance of the lung, *E_stat,RS_* static elastance of the respiratory system, *IAP* intraabdominal pressure, *PBW* predicted body weight, *P_driv_* driving pressure, *PEEP* positive end-expiratory pressure, *PEEP_ARDSNetwork_* PEEP titrated according to the ARDS Network lower PEEP table, *PEEP_Estat,RS_* PEEP titrated according to the lowest elastance of the respiratory system, *PEEP_Ptpexp_* PEEP titrated according to end-expiratory transpulmonary pressure, *Pes_exp_* esophageal pressure at positive end-expiratory pressure, *Pes_insp_* esophageal pressure at plateau airway pressure, *P_mean,RS_* mean airway pressure of the respiratory system, *P_peak,RS_* peak airway pressure of the respiratory system, *P_plat_* end-inspiratory plateau airway pressure, *Ptp_driv_* transpulmonary driving pressure, *Ptp_exp_* end-expiratory transpulmonary pressure, *Ptp_insp_* end-inspiratory transpulmonary pressure, *RR* respiratory rate, *V_D_/V_T_* ratio of physiologic dead space to tidal volume, *V_T_* tidal volume

**Table S9.** Gas exchange and hemodynamics of three PEEP titration strategies during supine and prone positioning

|  | **PEEP_ARDSNetwork_** | |  | **PEEP_Estat,RS_** | |  | **PEEP_Ptpexp_** | |  |
| --- | --- | --- | --- | --- | --- | --- | --- | --- | --- |
|  | **Supine** | **Prone** | ***p* value** | **Supine** | **Prone** | ***p* value** | **Supine** | **Prone** | ***p* value** |
| **PaO_2_/FiO_2_ (mm Hg)** | 136 ± 36 | 228 ± 86 | **<0.001** | 170 ± 72 | 237 ± 91 | **0.002** | 192 ± 76 | 240 ± 100 | **0.002** |
| **PaCO_2_ (mm Hg)** | 57.0 ± 10.5 | 57.9 ± 10.5 | 0.149 | 57.6 ± 10.3 | 57.7 ± 10.7 | 0.808 | 57.6 ± 10.4 | 58.0 ± 10.4 | 0.415 |
| **pHa** | 7.3 ± 0.1 | 7.3 ± 0.1 | 0.687 | 7.3 ± 0.1 | 7.3 ± 0.1 | 0.410 | 7.3 ± 0.1 | 7.3 ± 0.1 | 0.739 |
| **HR (beats/min)** | 92.6 ± 19.1 | 93.1 ± 21.2 | 0.746 | 92.8 ± 20.5 | 93.2 ± 21.4 | 0.411 | 92.6 ± 19.2 | 92.4 ± 20.6 | 0.869 |
| **MAP (mm Hg)** | 83.0 ± 10.8 | 87.1 ± 11.2 | **0.005** | 82.6 ± 9.8 | 89.5 ± 11.9 | **<0.001** | 79.2 ± 11.3 | 87.6 ± 11.4 | **<0.001** |
| **Noradrenaline (µg/kg/min)** | 0.2 ± 0.3 | 0.2 ± 0.3 | 0.117 | 0.2 ± 0.3 | 0.2 ± 0.3 | 0.222 | 0.2 ± 0.2 | 0.2 ± 0.2 | 0.409 |
| **CVP (mm Hg)** | 14.1 ± 6.4 | 16.8 ± 5.6 | **0.006** | 15.1 ± 6.9 | 16.8 ± 6.2 | 0.100 | 16.4 ± 7.4 | 17.5 ± 6.8 | 0.317 |
| **S_cv_O_2_ (%)** | 75.3 ± 7.4 | 81.2 ± 6.8 | **<0.001** | 77.6 ± 5.2 | 82.8 ± 6.2 | **<0.001** | 77.6 ± 6.1 | 81.4 ± 6.3 | **0.004** |
| **CI (L/min/m^2^)** | 3.7 ± 1.0 | 3.7 ± 0.9 | 0.683 | 3.5 ± 0.9 | 3.7 ± 1.0 | **0.021** | 3.2 ± 0.7 | 3.6 ± 0.8 | **<0.001** |

Values are means ± standard deviation of 40 patients with moderate to severe ARDS. Repeated measures ANOVA followed by Holm-Sidak’s post hoc test was used to compare the effects of different PEEP titration strategies on gas exchange and hemodynamics during supine and prone positioning (*p* < 0.05).

*CI* cardiac index, *CVP* central venous pressure, *HR* heart rate, *MAP* mean arterial pressure, *PaCO_2_* arterial partial pressure of carbon dioxide, *PEEP* positive end-expiratory pressure, *PEEP_ARDSNetwork_* PEEP titrated according to the ARDS Network lower PEEP table, *PEEP_Estat,RS_* PEEP titrated according to the lowest elastance of the respiratory system, *PEEP_Ptpexp_* PEEP titrated according to end-expiratory transpulmonary pressure, *PaO_2_/FiO_2_* arterial oxygen partial pressure divided by the fraction of inspired oxygen, *pHa* negative logarithm of the molar concentration of dissolved hydronium ions in arterial blood, *S_cv_O_2_* central venous oxygen saturation

**References**

1. Acute Respiratory Distress Syndrome Network, Brower RG, Matthay MA, Morris A, Schoenfeld D, Thompson BT, et al. Ventilation with lower tidal volumes as compared with traditional tidal volumes for acute lung injury and the acute respiratory distress syndrome. N Engl J Med. 2000;342(18):1301-8.

2. Beitler JR, Sarge T, Banner-Goodspeed VM, Gong MN, Cook D, Novack V, et al. Effect of Titrating Positive End-Expiratory Pressure (PEEP) With an Esophageal Pressure-Guided Strategy vs an Empirical High PEEP-Fio2 Strategy on Death and Days Free From Mechanical Ventilation Among Patients With Acute Respiratory Distress Syndrome: A Randomized Clinical Trial. JAMA. 2019;321(9):846-57.

3. Costa ELV, Slutsky A, Brochard LJ, Brower R, Serpa-Neto A, Cavalcanti AB, et al. Ventilatory Variables and Mechanical Power in Patients with Acute Respiratory Distress Syndrome. Am J Respir Crit Care Med. 2021.

4. Sinha P, Calfee CS, Beitler JR, Soni N, Ho K, Matthay MA, et al. Physiologic Analysis and Clinical Performance of the Ventilatory Ratio in Acute Respiratory Distress Syndrome. Am J Respir Crit Care Med. 2019;199(3):333-41.
